# Correction: Surface modification of intraocular lenses with hyaluronic acid and lysozyme for the prevention of endophthalmitis and posterior capsule opacification

**DOI:** 10.1039/d2ra90020c

**Published:** 2022-03-07

**Authors:** Bailiang Wang, Quankui Lin, Tingwei Jin, Chenghui Shen, Junmei Tang, Yuemei Han, Hao Chen

**Affiliations:** School of Ophthalmology & Optometry, Eye Hospital, Wenzhou Medical University Wenzhou 325027 China lqk97531@126.com Chenhao823@mail.eye.ac.cn +86 577 88067962; Wenzhou Institute of Biomaterials and Engineering, Chinese Academy of Sciences Wenzhou 32500 China; Department of Basic Teaching, City College of Wenzhou University Wenzhou 325027 China

## Abstract

Correction for ‘Surface modification of intraocular lenses with hyaluronic acid and lysozyme for the prevention of endophthalmitis and posterior capsule opacification’ by Bailiang Wang *et al.*, *RSC Adv.*, 2015, **5**, 3597–3604, DOI: 10.1039/c4ra13499k.

The authors regret to inform that, the representative images of waterborne *S. aureus* adhesion on pristine PMMA, HA-5% lysozyme in Fig. 2 and growth and morphology of HLECs adhesion on TCPS were incorrectly marked (Fig. 2a, 2c and 6a, respectively). The corrected versions are shown below. The correction does not change any description, results or conclusions of the original paper.

**Fig. 2:**
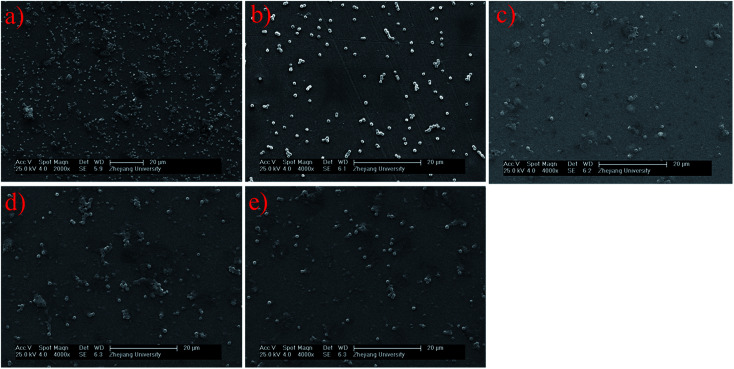
SEM images of (a) pristine PMMA and (b) HA, (c) HA-5% lysozyme, (d) HA-10% lysozyme, (e) HA-20% lysozyme coated on PMMA after exposure to waterborne *S. aureus*.

**Fig. 6:**
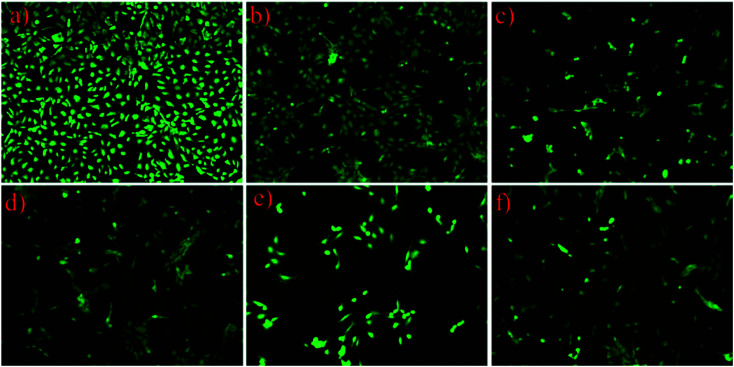
Growth and morphology of HLECs stained with FDA after 24 h of incubation on (a) TCPS, (b) pristine PMMA, (c) HA, (d) HA-5% lysozyme, (e) HA-10% lysozyme and (f) HA-20% lysozyme, under fluorescence microscopy (the magnification is 10×).

The Royal Society of Chemistry apologises for these errors and any consequent inconvenience to authors and readers.

## Supplementary Material

